# Dispensing practices of amoxicillin suspension by community pharmacists

**DOI:** 10.1017/ash.2024.360

**Published:** 2024-09-03

**Authors:** Preeti Jaggi, Latania Logan

**Affiliations:** Emory University and Children’s Healthcare of Atlanta, Atlanta, Georgia

## Abstract

**Background:**

Amoxicillin suspension is frequently prescribed to children; we hypothesized that prescribing convention system constraints lead to unusual dosing regimens and unnecessary waste of the drug.

**Objective:**

Identify antibiotic dispensing practices by community pharmacists and/or technicians to understand opportunities to decrease wasted amoxicillin liquid and optimize prescribing convention of liquid amoxicillin to children.

**Methods:**

Pilot online survey of Atlanta area and National Community Pharmacists Association pharmacists or pharmacy technicians that self-reported dispensing amoxicillin suspension. Questions regarding liquid amoxicillin dispensing practices and other open comments were asked about suggestions to decrease amoxicillin waste from March 13 to April 5, 2023.

**Results:**

Among 68 pharmacy staff that participated, over 90% reported dispensing extra liquid amoxicillin to patients for more than 10% of the doses they dispensed. Twenty-seven respondents (39.7%) felt that amoxicillin waste was a problem; waste was most often due to package/bottle sizing issues (n = 64 of 67 responses, 95.5%). Respondents reported instructing families to dispose of extra medication in the trash (n = 51, 75%); 11 (16.2%) instructed pour the remaining in the sink; none reported requesting return to the pharmacy, and 6 (8.8%) reported other instructions. Community pharmacists observed that computerized algorithms create odd dosing amounts and that some prescribers add to the overall amount needed routinely.

**Conclusion:**

Community pharmacists in this pilot survey observed prescribing conventions, manufacturing, regulatory, and electronic medical record constraints that lead to liquid amoxicillin waste or confusing amounts for families to use.

## Introduction

Amoxicillin is the most widely prescribed antibiotic to children with an estimated 39.3 million prescriptions dispensed each year.^1^ Recently, amoxicillin shortages have caused limited availability of this drug.^2^ When this occurs, broader drugs are recommended, and these may lead to decreased quality of life indicators in children.^3,4^ We hypothesized that systemic issues in prescribing conventions and availability cause unnecessary amoxicillin suspension waste to occur and can lead to confusion for parents in administering the drug to children. We sought to identify the antibiotic dispensing practices by community pharmacy staff to understand opportunities to decrease amoxicillin liquid wasted into the environment. In addition, we sought to solicit from pharmacists other opportunities to improve prescribing convention of liquid amoxicillin to children.

## Methods

With IRB approval, we electronically surveyed metropolitan Atlanta area community pharmacists (survey in Supplement 1) and/or pharmacists/pharmacy technicians from the National Community Pharmacists Association regarding liquid amoxicillin dispensing habits and other open comments about suggestions to decrease amoxicillin waste from March 13th to April 5, 2023. The National Community Pharmacists Association represents independent, community pharmacists. Respondent answers were excluded if the respondent indicated that they were not a pharmacist/technician and if they initially self-reported that they “do not dispense liquid amoxicillin”.

## Results

*Survey distribution*. The survey was sent through informal connections of metropolitan Atlanta area pharmacists. In addition, the survey was sent to an electronic database via email of industry stakeholders and community pharmacists through the National Community Pharmacists Association (NCPA). There are approximately 1000 receiving newsletters from NCPH, but this also includes corporate members and other industry stakeholders and exact number of pharmacists in this group was not available from NCPA. Among participants, 68 responses were eligible for analysis because they reported that they regularly dispense liquid amoxicillin. Respondents were from 18 states with the majority being from Georgia (n = 39, 57.4%). The majority were female (n = 44, 64.7%). More than half of respondents reported practicing for 21 or more years (n = 35, 51.5%), followed by 11–20 years (n = 16, 23.5%). Reported estimation of excess liquid amoxicillin dispenses can be found in Table 1. Respondents reported instructing families to dispose of extra medication in the trash (n = 51, 75%); 11 (16.2%) instructed pour the remaining in the sink; none reported requesting return to the pharmacy, and 6 (8.8%) reported other instructions. Twenty-seven respondents (39.7%) felt that amoxicillin waste was a problem; waste was most often due to package/bottle sizing restraints (n = 64 of 67 responses, 95.5%). The remainder cited insurance preference for bottle size coverage as the reason. Complete list of responses can be found in Supplemental Table 2. Themes that were observed to minimize liquid amoxicillin waste with representative comments are provided in Table 2.

**Table 1.** Pharmacist/pharmacy technician responses about liquid amoxicillin waste practices

Estimated percentage of dispensations by pharmacist	<10%	10–19%	20–30%	31–50%	>50%
	Number of Responses, (percentage of responses)				
How often do you dispense more volume of amoxicillin than is written for? n, (%)	5, (7.4%)	2 (2.9%)	14, (20.6%)	19 (27.9%)	28 (41.2%)
How often do you instruct families for what to do with any extra leftover amoxicillin? n, (%)	2, (2.9%)	0, (0.0%)	4, (5.9%)	5, (7.4%)	57, (83.8%)
What percent of time do you dispense a cup or syringe with the amoxicillin suspension? n, (%)	1, (1.5%)	2, (2.9%)	0, (0.0%)	2, (2.9%)	63, (92.6%)

**Table 2.** Themes and representative comments regarding amoxicillin dispensation

Theme	Do you have any suggestions for how to conserve oral amoxicillin suspension?
Computerized algorithms create odd amounts	“Take away computerized dosing algorithms based on precise patient weight. Instead, dispense staggered (“stair step”) dosage based on available package sizes.”
Prescribing conventions	“some practices routinely write for 10ml extra than what is needed for the course of treatment due to spillage, which causes more problems. For example, if 200 mL is needed and they prescribe 210 mL…then we then have to dispense 250ml or 300ml” “prescribers should look at the final amount they are trying to prescribe and decide in their professional opinion if the treatment could be reduced by a day or so to not waste another whole bottle”
Regulatory constraints	“If manufacturers could make a shelf stable suspension (think like ibuprofen suspension) then we can pour exact amount needed into dispensing bottles as opposed to having to give 2 bottles of 100ml amoxicillin for a kid who only needs 160ml for the course of treatment” “Make bulk amoxicillin powder commercially available to be weighed by the pharmacist for reconstitution” “The laws on rounding are not as clear for retail pharmacists as they are in hospital. Hospital pharmacists have institution policies that allow for plus or minus 10% rounding for pediatrics. We don’t have that in retail pharmacy and we don’t have the time to fix every prescription that we get.”

## Discussion

Community pharmacists in this survey observed prescribing conventions, manufacturing, regulatory, and electronic medical record constraints that lead to liquid amoxicillin waste or confusing amounts for families to use. Antibiotic contamination of the soil or water may increase antibiotic-resistant bacteria and represents potential sources of human and animal exposure. Antibiotics in surface water and soil have been detected across the U.S. and directly impact groundwater and drinking/recreational water sources in urban areas.^5^ Antibiotics placed down drains can further propagate antibiotic resistance among persistent bacterial communities associated with biofilms and water stagnation in plumbing systems.^6,7^

Considerations to minimize liquid amoxicillin waste include chewable amoxicillin tablets, which are available in a generic format, considered safe down to two years of age,^8^ and would result in decreased antibiotic waste and plastic waste and avoids the need for refrigeration. Chewable tablets can be provided precisely without concern of spillage. Avoiding extra amounts of liquid can both avoid confusion for families and decreases the likelihood that families will use the antibiotic later inappropriately.^9^ Finally, regulators and manufacturers could consider providing bulk powder to pharmacies for reconstitution so that the patient-specific amount can be dispensed each time. Another opportunity for modification of regulations would be to allow the pharmacist to round for bounded, short-duration antibiotic courses to preserve antibiotic stockpiles and minimize waste. Infectious diseases societies could consider advocacy to change these types of regulation.

Another area that community pharmacists noted was that many prescriptions are very specific, such as, for example, 7.2 mL of amoxicillin to be given per dose. Errors in dispensing liquid medication by parents are not uncommon, noted as up to 40% in one study of 287 family units.^10^ Avoiding this type of hyper precise dosing could be undertaken by electronic medical record informaticists with the help of clinical pharmacists to use standard doses for weight ranges. Dosing for common upper respiratory tract infections for amoxicillin ranges from 70 to 90 mg/kg/day for acute otitis media, pneumonia, and sinusitis, so provides some flexibility in the amount to be dispensed.

Limitations of this study include that it mostly represented pharmacists practicing in Georgia and independent pharmacy personnel, introducing potential for bias. There was an assumed low response rate (exact rate was unable to be calculated), which limits the generalizability of this study. These can be considered preliminary data that could be confirmed by a larger survey.

## Conclusion

There are several modifiable systems that contribute to unnecessary liquid amoxicillin waste and inconvenient doses that prescribers can optimize by changing prescribing convention.

## Supporting information

Jaggi and Logan supplementary material 1 Jaggi and Logan supplementary material

Jaggi and Logan supplementary material 2 Jaggi and Logan supplementary material

## Supplementary material

The supplementary material for this article can be found at https://doi.org/10.1017/ash.2024.360.
