# Supplementary material for: Dispensing practices of amoxicillin suspension by community pharmacists
Source: Antimicrob Steward Healthc Epidemiol. 2024 Sep 3;4(1):e112. doi: 10.1017/ash.2024.360 (PMC11736456; doi:10.1017/ash.2024.360)
Supplement: Jaggi and Logan supplementary material 1 — Jaggi and Logan supplementary material [file S2732494X24003607sup001.pdf]

Dispensing Practices

Please complete the survey below.

Thank you!

Survey Date (auto populates, not seen by outside user)

____

Are you a licensed pharmacist or a pharmacy technician in the outpatient setting?

◯ Yes
◯ No

Do you dispense oral amoxicillin suspension?

◯ Yes
◯ No

How often do you dispense more volume of amoxicillin than is written for?

◯ < 10% of oral suspenions I dispense
◯ 10-19% of oral suspensions I dispense
◯ 20-30% of suspensions I dispense
◯ 31-50% of suspensions I dispense
◯ >50% of suspensions I dispense

How often do you instruct patients/families about what to do with any extra amoxicillin that might be leftover?

◯ < 10% of oral suspenions I dispense
◯ 10-19% of oral suspensions I dispense
◯ 20-30% of suspensions I dispense
◯ 31-50% of suspensions I dispense
◯ >50% of suspensions I dispense

What do you generally instruct them to do with the extra liquid medication?

◯ Throw it in the trash
◯ Return to the pharmacy
◯ Pour down the sink
◯ Other

Please specify other instruction you advise

____

What percent of the time do you dispense a syringe or cup with the amoxicillin suspension?

◯ < 10% of oral suspenions I dispense
◯ 10-19% of oral suspensions I dispense
◯ 20-30% of suspensions I dispense
◯ 31-50% of suspensions I dispense
◯ >50% of suspensions I dispense

In your personal practice, if you have ever dispensed more antibiotics than is prescribed, why is this practice done?

◯ Reconstitution of some drugs requires more volume
◯ In case there is spillage of medication
◯ Other reason
◯ I never dispense extra amoxicillin

Please specify other reason

____

In which state do you currently practice?

◯ Alabama
◯ Alaska
◯ Arizona
◯ Arkansas
◯ California
◯ Colorado
◯ Connecticut
◯ Delaware
◯ District of Columbia
◯ Florida
◯ Georgia
◯ Hawaii
◯ Idaho
◯ Illinois
◯ Indiana
◯ Iowa
◯ Kansas
◯ Kentucky
◯ Louisiana
◯ Maine
◯ Maryland
◯ Massachusetts
◯ Michigan
◯ Minnesota
◯ Mississippi
◯ Missouri
◯ Montana
◯ Nebraska
◯ Nevada
◯ New Hampshire
◯ New Jersey
◯ New Mexico
◯ New York
◯ North Carolina
◯ North Dakota
◯ Ohio
◯ Oklahoma
◯ Oregon
◯ Pennsylvania
◯ Puerto Rico
◯ Rhode Island
◯ South Carolina
◯ South Dakota
◯ Tennessee
◯ Texas
◯ Utah
◯ Vermont
◯ Virginia
◯ Virgin Islands
◯ Washington
◯ West Virginia
◯ Wisconsin
◯ Wyoming

What is your gender?

◯ Male
◯ Female
◯ Prefer not to answer

How long have you been practicing as a licensed pharmacist?

◯ 0-5 years
◯ 6-10 years
◯ 11-20 years
◯ 21 or more years

Do you perceive antibiotic suspension waste as a problem?

◯ Yes
◯ No

Do you have any suggestions for how to conserve oral amoxicillin suspension (free text)
