# Supplementary material for: Dispensing practices of amoxicillin suspension by community pharmacists
Source: Antimicrob Steward Healthc Epidemiol. 2024 Sep 3;4(1):e112. doi: 10.1017/ash.2024.360 (PMC11736456; doi:10.1017/ash.2024.360)
Supplement: Jaggi and Logan supplementary material 2 — Jaggi and Logan supplementary material [file S2732494X24003607sup002.docx]

Supplemental Table 2

| **Do you have any suggestions for how to conserve oral amoxicillin suspension?** |
| --- |
| PBMs require that we dispense stock size that we bill for. |
| The most waste and haste to the patient and pharmacy from a single script is due to long duration of use, especially if small volumes, something along the lines of. "Give 2.5mL po x14 days, then discard remainder and return to pharmacy for additional bottle." This is a rare instance, but is the only time I personally have been truly concerned with the amoxicillin suspension waste. |
| Prescribing by weight is now common and odd amounts above 1-2 teaspoonfuls are prescribed that end up leading to odd total volumes needed for a full course.  this leads to the most waste and creates anxiety amongst parents trying to measure these amounts such as 6.1 ml.  prescribers should stick to amounts such as 1/2, 1, 1&1/2 teaspoonfuls and less waste would occur. |
| Making more sizes of amoxicillin suspension would be helpful. That way we could dispense closer to the amount needed and not have to dispense way more than needed. |
| We stock multiple sizes of amoxicillin in each strength so that we can get to the closest dispensed amount. For the amoxicillin that are dispensed for a larger quantity than written, the waste amount is typically less than 30 mL's (average of 26 mL in the month sample I looked at). However, in our sample 80% of the amoxicillin dispensed fall in this category. |
| Since prescribers are writing for more precise dosing/weight, there will be waste in commercial reconstituted medications. |
| Kits that allow for the correct amount to be dispensed. |
| Look at other therapies that may be available.   Convert dosing to use a smaller package size. |
| More package sizes to choose from. With prescriber EHR systems calculating dose based on exact waste most rx's aren't written for commercially available package sizes. |
| pharmacists should be allowed to use multiple package sizes, and bill for multiple sizes to reduce the amount wasted (i.e. using a 50ml and 75ml bottles to get to 125ml, instead of insurance forcing us to use (2) 75ml bottles) |
| 1) allow the pharmacist to adjust the dose to the lowest commercially available volume when appropriate. Many times we can tell someone other than the physician entered the order when the dose has decimals used in the volume to be given per dose (example: 7.8ml bid).  2) make bulk amoxicillin powder commercially available to be weighed by the pharmacist for reconstitution of customized volumes for specific patients. |
| Have a broader range of Qty available. |
| I dispense the package size that will take care of the full day supply prescribed. For example, If the patient has a dose of 12.5ml BID for 10 days, the patient will need 250 mL. At the moment my pharmacy stocks 100 ml bottles and 75 ml bottles (due to shortages). I end up having to dispense 300ml and the patient wastes 50ml. Due to stability the drug cannot be stored in bulk as a liquid suspension. Most pharmacies can't weigh powders to make custom package sizes. I know Amoxil is a weight based dose, but could the suspension dosing not be more standardized like the capsule dosing (increments of 500 and 250? A lot of issue is the often extremely specific dosing for each patient. |
| Prescribing only when appropriate |
| have a bulk container to dispense from.would be messy and time consuming |
| Take away computerized dosing algorithms based on precise patient weight. Instead, dispense staggered ("stair step") dosage based on available package sizes. ***BUT MOST OF ALL - STOP PRESCRIBING UNNECESSARY ANTIBIOTICS FOR VIRAL INFECTIONS*** |
| I know that antibiotic suspension waste is a major problem. However, the only solutions I could think of would be to find a way to make a stable suspension or solution that involves most oral liquid antibiotics (i.e. Sulfatrim suspension) or to use an IV or IM formulation (i.e. Rocephin). |
| More variation is pack size availability |
| If manufacturers could make a shelf stable suspension (think like ibuprofen susp) then we can pour exact amount needed into dispensing bottles as opposed to having to give 2 bottles of 100ml amox for a kid who only needs 160ml for the course of treatment.   there are also pediatric practices (an example is Peachtree Park pediatrics in Atlanta) that routinely write for 10ml extra than what is needed for the course of treatment due to spillage, which causes more problems. example 10ml bid x10 days written for 210ml instead of 200ml actually needed. we then have to dispense 250ml or 300ml depending on what pack sizes are in stock |
| no as all patients come in different ages and sizes and a dose can be all over the spectrum in children. this also applies to any other mix type antibiotics as well |
| If the physician looks at the sizes Amoxil comes in and prescribe accordingly, it will save a lot of trouble for the pharmacist. I have seen a dose of 1.344 ml.  For a volume so precise, it is nearly impossible for the patient to measure it out. |
| Have the government pass a law forbidding people to be sick. |
| Be aware of available package sizes |
| Most of the waste has occurred when we have only one or two bottle presentations available in the pharmacy (due to the shortage). Also, the use of EMR prescribing, although it is weight-based, results in an odd dose for the parent to measure (e.g., 10.25 ml). This can make the difference in bottle size  The laws on rounding are not as clear for retail pharmacists as they are in hospital. Hospital pharmacists have institution policies that allow for +/- 10% rounding for pediatrics. We don't have that in retail pharmacy and we don't have the time to fix every prescription that we get. Our technician  staff has been cut back to unsafe levels already. We're doing the best we can just to get the medication dispensed as written. |
| instead of formulating them to be reconstitutions, make it be dispensable in bulk, so we can fill for exactly the amount needed for the patient and nothing gets wasted. |
| In general, I think the over prescribing of antibiotics is a problem, particularly when many are prescribed for viral illnesses.  Historically, antibiotic waste has not been a problem until this year when we saw an increase in viral infections (RSV, Flu, COVID, traditional coronavirus-causing "colds") and the over-prescribing of antibiotics related to that surge.  Seems to me that a combination of improperly prescribing antibiotics with a surge of viral upper respiratory infections contributed to the shortage. |
| Prescribers should look at the final amount they are trying to prescribe and decide in their professional opinion if the treatment could be reduced by a day or so to not waste another whole bottle since the bottles come prepackaged. |
| Doctors need to prescribe accurate doses so patient is getting the right dose for the right indication.  Doctors often way over prescribe for the indication. Some sort of news letter or email about the amoxicillin shortage needed to inform doctors about cutting back on doses in order to prevent over medication and shortages of the antibiotic |
| Prescriber to use dosing range to dose based on how it comes packaged. |
| According to current USP 795 standards, any water-containing reconstituted suspension has a maximum Beyond Use Date of 14 days with refrigeration. The commercially-available package sizes are 50, 100 or 150ml. Not all patient-specific dosing regimens will use an entire bottle. The manufacturer could potentially use an alternative suspending agent (non aqueous) to commercially produce a product with a longer shelf life. |
